# The Applicability of Die Cast A356 Alloy to Additive Friction Stir Deposition at Various Feeding Speeds

**DOI:** 10.3390/ma14206018

**Published:** 2021-10-13

**Authors:** Bandar Alzahrani, Mohamed M. El-Sayed Seleman, Mohamed M. Z. Ahmed, Ebtessam Elfishawy, Adham M. Z. Ahmed, Kamel Touileb, Nabil Jouini, Mohamed I. A. Habba

**Affiliations:** 1 Mechanical Engineering Department, College of Engineering at Al Kharj, Prince Sattam Bin Abdulaziz University, Al Kharj 16273, Saudi Arabia; ba.alzahrani@psau.edu.sa (B.A.); k.touileb@psau.edu.sa (K.T.); n.jouini@psau.edu.sa (N.J.); 2 Metallurgical and Materials Engineering Department, Faculty of Petroleum and Mining Engineering, Suez University, Suez 43512, Egypt; mohamed.elnagar@suezuniv.edu.eg (M.M.E.-S.S.); ebtessam.elfishawy@aucegypt.edu (E.E.); 3 Metallurgical and Materials Engineering Department, Faculty of Engineering, Karabuk University, Karabuk 78050, Turkey; adhamzaky305@gmail.com; 4 Mechanical Department, Faculty of Technology & Education, Suez University, Suez 43518, Egypt; Mohamed.Atia@suezuniv.edu.eg

**Keywords:** additive friction stir deposition, A356, feeding speeds, microstructure and hardness

## Abstract

In the current investigation, additive friction stir-deposition (AFS-D) of as-cast hypoeutectic A356 Al alloy was conducted. The effect of feeding speeds of 3, 4, and 5 mm/min at a constant rotational speed of 1200 rpm on the macrostructure, microstructure, and hardness of the additive manufacturing parts (AMPs) was investigated. Various techniques (OM, SEM, and XRD) were used to evaluate grain microstructure, presence phases, and intermetallics for the as-cast material and the AMPs. The results showed that the friction stir deposition technique successfully produced sound additive manufactured parts at all the applied feeding speeds. The friction stir deposition process significantly improved the microstructure of the as-cast alloy by eliminating porosity and refining the dendritic α-Al grains, eutectic Si phase, and the primary Si plates in addition to intermetallic fragmentation. The mean values of the grain size of the produced AMPs at the feeding speeds of 3, 4, and 5 mm/min were 0.62 ± 0.1, 1.54 ± 0.2, and 2.40 ± 0.15 µm, respectively, compared to the grain size value of 30.85 ± 2 for the as-cast alloy. The AMPs exhibited higher hardness values than the as-cast A356 alloy. The as-cast A356 alloy showed highly scattered hardness values between 55 and 75.8 VHN. The AMP fabricated at a 3 mm/min feeding speed exhibited the maximum hardness values between 88 and 98.1 VHN.

## 1. Introduction

Nowadays, due to the need for specific and advanced engineering materials for particular applications, additive manufacturing (AM) technology is growing very fast, and in the near future, it is thought it will occupy most industrial sectors. This technology is a more suitable method to produce metallic parts for the automobile and aircraft industries [1,2,3]. Among the AM techniques [4], friction stir deposition (FSD) is used instead of fusion-based techniques [5] in the manufacturing of aluminum alloys [6,7], copper alloys [8], and steel [9]. This solid-state metal AM technology is based on friction stirring principles [10,11,12], adding a mechanism of material feeding. In most recent studies, it has been called additive friction stir deposition (AFSD) [13]. AFSD, as a thermomechanical process, is typically similar to friction stir processing [11,14,15,16], and welding [17,18,19] in heat generation, heat dissipation, and heat transfer mechanisms in the stir zone. The thermal stresses in the additive manufactured part (AMP) produced by the AFSD process are likely to be minimal, owing to a lower temperature gradient when compared to additive processes involving melting and solidification [2]. The mechanism of AFSD consists of a consumable rod to deliver the feed material directly to a fixed substrate plate. The rapid rotation of the rod against the substrate generates friction heat at the interface between the rod and substrate plate. Due to the frictional heat, the feed material is softened and plastically deformed, which bonds to the substrate. Continuous bonding of more plastically deformed feed material to the substrate plate makes the deposition of the first layer, and subsequently, the addition of layers one by one makes a 3D object of a desired thickness [20,21,22]. Dilip et al. [23] studied the microstructure features of fabricated AA2014-T6 by AFSD at 800 rpm, 1 mm/min, at an axial pressure of 35 MPa. The microstructure features were very fine grains and refined second-phase precipitates. Perry et al. [24] reported that the AA2024 friction stir deposited at 300 rpm rotational speed, 2 mm/s traverse speed, and feed rate of 0.85 mm/s shows an almost fully recrystallized microstructure. Priedeman et al. [8] investigated the microstructure and mechanical properties of AFSD 110 Cu alloy processed at a rotational speed of 275 rpm and a traverse speed of 2.12 mm/s. The results indicated that the microstructure appeared to be recrystallized grains throughout the deposit layers, with different degrees of refinement, and the hardness value revealed that the deposited material was softer than that of the starting feedstock. Dilip et al. [25] succeeded in depositing individual layers 1–2 mm up to a height of 30 mm of austenitic stainless steel AISI 304 via the FD process. The obtained microstructure revealed an equiaxed fine-grained microstructure and the grain refining was ascribed to dynamically recrystallization. The growing demands for reducing energy consumption and producing more fuel-efficient vehicles are a big challenge. Cast A356 (hypoeutectic Al alloy containing 6.5–7.5% Si) is widely used for manufacturing aerospace and automotive components due to its high strength-to-weight ratio, good castability, good mechanical properties, and good corrosion resistance [26,27]. Despite several researchers [28,29,30,31] studing the properties and processing parameters to deposit different Al alloys using solid-state techniques, there is no publication discussing the AFSD of cast A356 alloy. Thus, this work explores the applicability of producing additive manufacturing parts (AMPs) from the die-cast A356 (Al-7.3%Si) alloy using AFSD at different feeding rates of 3, 4, and 5 mm/mins. The microstructure features and hardness of the produced AMP materials as compared to the staring feedstock are discussed.

## 2. Methodology

### 2.1. Starting Materials

**Figure 1:**
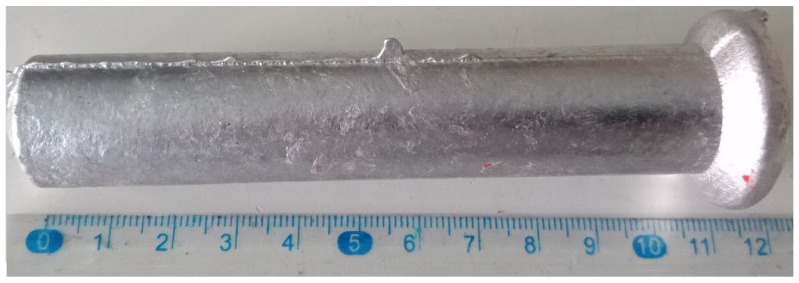
The consumable rod of the as-cast Al-Si before the machining process.

The starting material used in the current work is as-cast A356 aluminum alloy fabricated by the die-casting technique. The rod dimensions were 22 mm diameter and 130 mm length. The as-cast rods were machined to the final dimensions of 20 mm diameter and 100 mm length (Figure 1).

**Figure 2:**
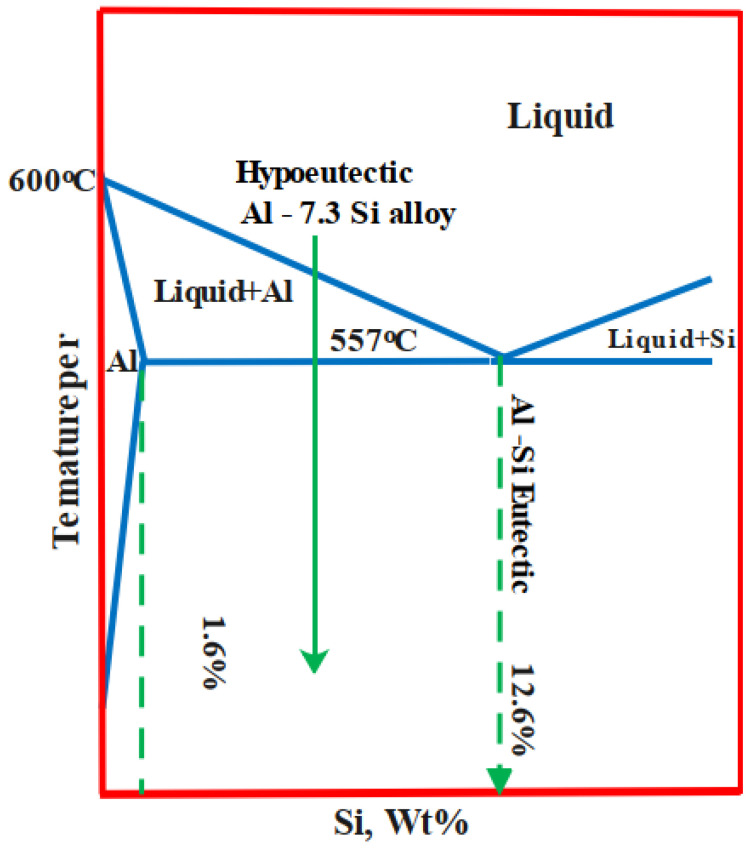
Sketch showing part of the Al-Si binary phase diagram.

The chemical composition of the as-cast A356 was investigated using Foundry-Master pro, (Oxford Instruments, Abingdon, UK). Table 1 lists the chemical composition of the as-cast A356 alloy. It can be seen that the major alloying elements in the Al base are Si, Cu, and Fe, with traces of other elements including Zn, Mg, Sn, and Mn. Figure 2 illustrates part of the Al-Si binary phase diagram showing the A365 alloy.

### 2.2. AM Process and Evaluation of the Produced Materials

**Figure 3:**
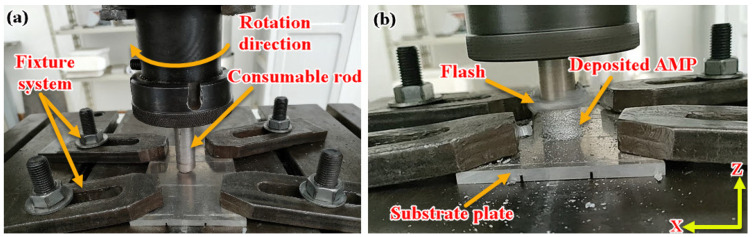
Photo images showing (**a**) fixing the A356 consumable rod and substrate AA2024 on the FSW/FSP machine, (**b**) the process features of the AFS-D A356 material.

In order to study the feasibility of the AFSD of A356 (hypoeutectic Al-7.3Si) alloy, three continuous-deposited multi-layers were produced at a constant rotation rate of 1200 rpm and different feeding speeds of 3, 4, and 5 mm/min using the consumable rod of A356 alloy. The AFSD process was performed using a full-automatic friction stir welding machine (EG-FSW-M1) [32,33]. Before starting the deposition process, the consumable A356 rods were gripped to the machine shank, and the aluminum substrate was fixed on the machine table. Once the process began, the consumable rod tip was brought down to touch the substrate surface (Figure 3a). Afterwards, the rod was rotated at a constant spindle rotation rate of 1200 rpm. Based on our lab experience, three different feeding speeds of 3, 4, and 5 mm/min were chosen and applied. Due to the severe friction between the rod and the fixed substrate during the AFSD process, the A356 consumable rod was exposed to the generated frictional heat. It was reported that the applied heat ranged from 0.6 to 0.9 of the melting point of the alloy [28,34]. This justifies the softening of the rod, which contributes to the material deposition from the rod to the substrate, forming the first deposited layer. While the process continued, more layers were deposited to form a built part, as given in Figure 3b.

### 2.3. Samples Preparation and Testing

**Figure 4:**
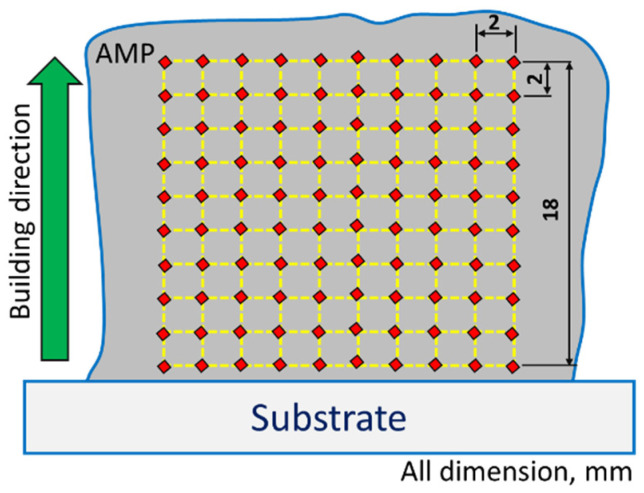
A schematic drawing illustrates the location of hardness mapping measurements.

The deposited parts were prepared for the microstructural examination and hardness evaluation by cross-sectioning across the deposition direction (z-direction), as shown in Figure 3b. A standard metallographic procedure was executed by cutting, mounting, grinding, and polishing to an 0.05 µm alumina surface finish. The microstructural features were subsequently revealed by etching for 2 min using Keller’s etching solution. Microstructural analysis and characterization were extensively carried out with an optical microscope (Olympus, BX41M-LED, Tokyo, Japan). Furthermore, the grain size analysis of the AMPs and the BM were measured using the line interception method in Olympus Stream Motion Software. In addition, scanning and EDS analysis was performed using a scanning electron microscope (SEM, Thermo-Scientific, Quattro S, Waltham, MA, USA) equipped with an AMETEK energy-dispersive spectroscope (EDS). For the base metal and one of the deposited parts, XRD analysis was performed using an X-ray diffractometer (Malvern PANALYTICAL, Empyrean, Almelo, The Netherlands) with a Molybdenum anode of a Kα wavelength of 0.07 nm, using High Score Plus software. Vickers microhardness measurements were carried out at a load of 200 g with holding time of 15 s to evaluate the average hardness of the as-cast and the AM built parts using a Vickers Hardness Tester (Qness Q10, Salzburg, Austria). The hardness maps were also drawn by collecting five vertical lines (parallel to building direction) with a 2 mm interval between every 2 points, as shown schematically in Figure 4.

## 3. Results and Discussion

### 3.1. Macrostructure Examination

**Figure 5:**
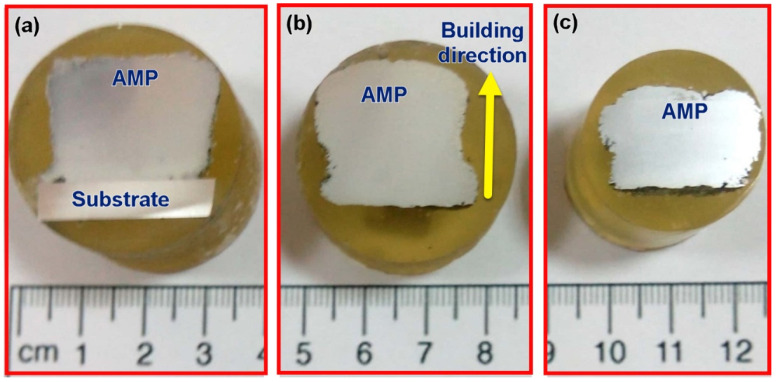
Cross-section macrographs of the produced A356 AMPs at 1200 rpm rotation rate and various feeding speeds (**a**) 3 mm/min, (**b**) 4 mm/min, and (**c**) 5 mm/min. Reproduced with copyright permission from Springer nature [20].

**Figure 6:**
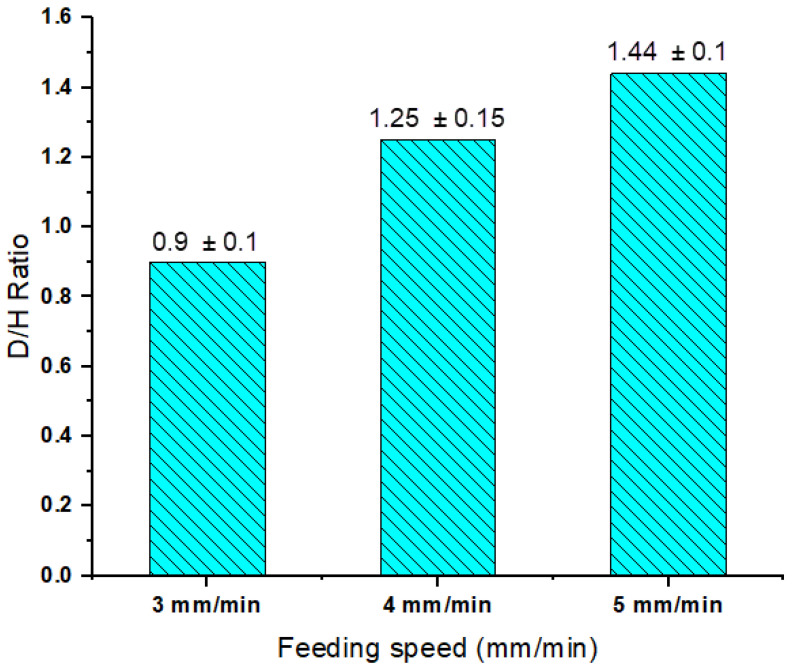
Diameter/height ratio of AMPs as a function of feeding speed after production at a constant rotation rate of 1200 rpm.

The macrostructure examination of AMPs produced at a constant rotation rate of 1200 rpm with feeding speeds of 3, 4, and 5 mm/min have revealed defect-free parts as can be seen in Figure 5 which shows the macrographs of the deposited parts. The visual examination of the deposit layers showed nearly no flash accumulated around the produced A356 AMPs at the applied processing parameters, which indicates a good choice of the rotational rate and the feeding speed. At all the processing parameters, the macrostructure cross-sections of the fabricated AMPs show fully continuous structures without any bonding defects at the interfaces of the deposited layers, Figure 5 and Figure 6 represent the effect of feeding speed on the diameter/height (D/H) ratios of the AMPs produced at a rotational speed of 1200 rpm. It can be observed that the D/H ratio of the fabricated AMPs increased with increasing feeding speeds from 3 to 5 mm/min. The as-cast A356 alloy plasticity during the AFS-D process was governed by the amount of heat input in the stir zone through the production process of the AMPs. The heat input in the AFS-D process is related mainly to rotational rate and feeding speed.

**Figure 7:**
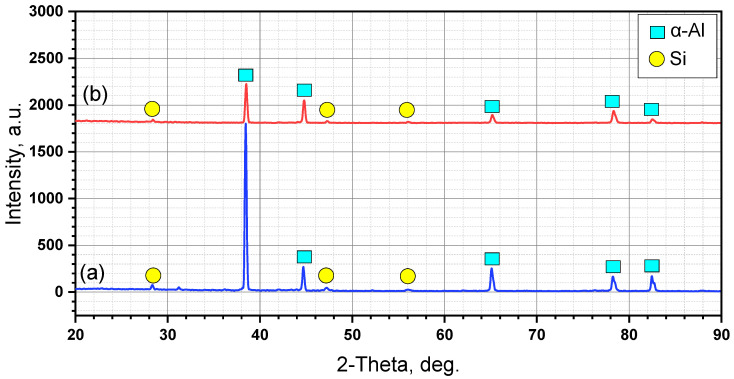
XRD patterns of (**a**) as-cast A356 and (**b**) AMP deposited at 1200 rpm and 3 mm/min.

XRD examination was carried out to determine the present phases and intermetallics of the as-cast A356 alloy and the AMPs. The obtained results show only the peaks of α-Al and primary Si phases for both the as-cast and AMP materials, as can be observed from the XRD chart in Figure 7. Due to the limitation of XRD detection of phases lower than 5%, no peaks appeared for the intermetallics in either the as-cast or AMP alloys. However, only little peak can be observed at a 2-theta of about 31, which might belong to the intermetallic but cannot be confirmed as it is only one peak.

### 3.2. Microstructure Examination

#### 3.2.1. Microstructure of the As-Cast A356

**Figure 8:**
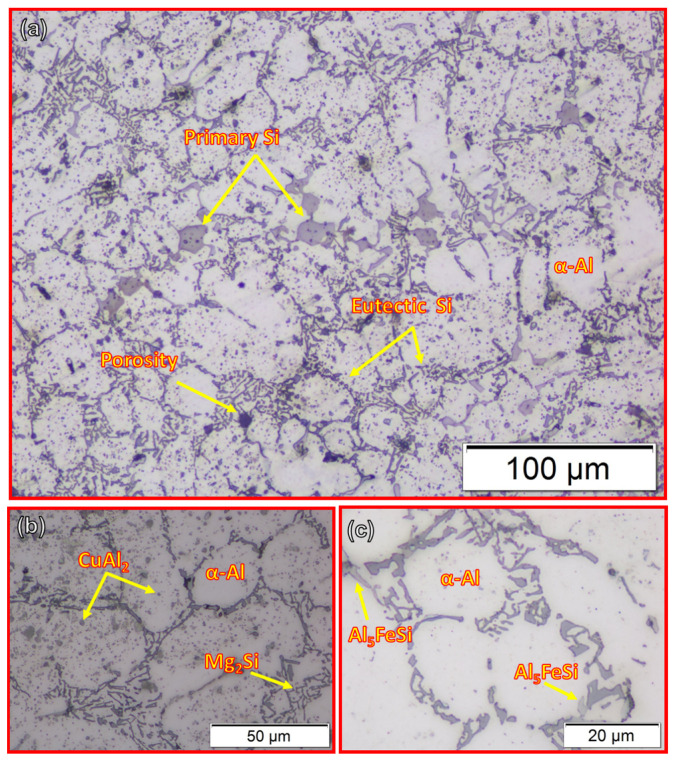
OM micrographs show the microstructure of (**a**) the as-cast A356 alloy, (**b**,**c**) at higher magnifications.

Figure 8 illustrates the microstructure of the as-cast A356 alloy. It can be seen that the microstructure consists of dendritic α-Al arms surrounded by a Si-rich eutectic phase, porosity, primary free-Si, and intermetallic phases such as almost spherical particles of light grey CuAl_2_, fishbone-like black Mg_2_Si, and light grey Fe-containing intermetallics (Al_5_FeSi) distributed at the grain boundaries of the α-Al matrix. The same microstructure features have been reported by other authors [35,36,37,38,39,40] for A356 alloys with little difference according to modifier addition [37,39,40] and casting techniques [38,40]. The average grain size of the as-cast material (Figure 8) is 30.85 ± 2 µm. Moreover, the dispersion of the primary free-Si is not uniform throughout the A356 matrix. The mean size of the primary Si is about 11.3 ± 0.5 µm.

#### 3.2.2. Microstructure of the Consumable Rod and A356 AMPs

**Figure 9:**
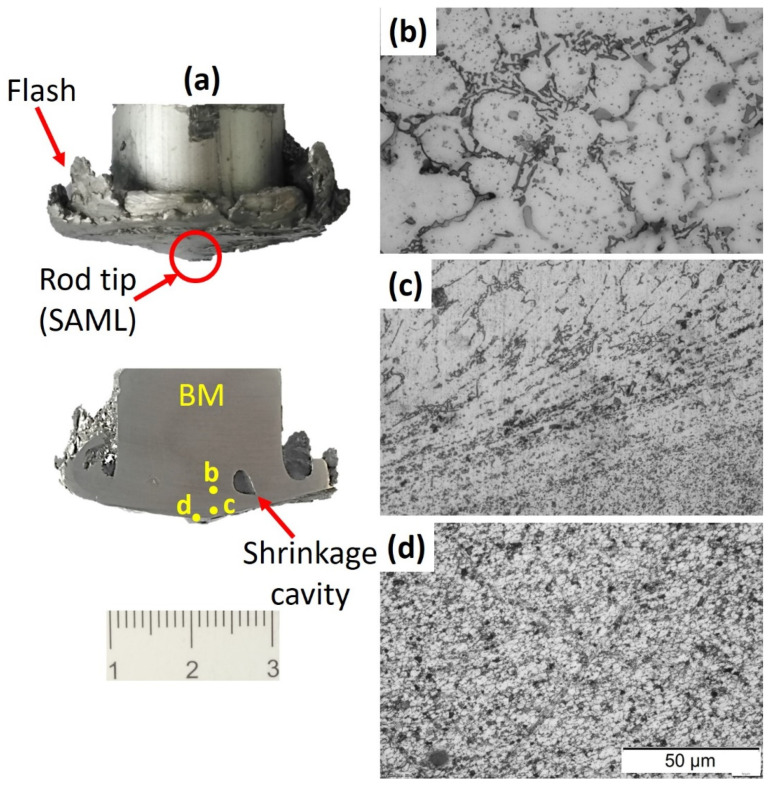
(**a**) Photograph of the remaining A356 consumable rod split halves, (**b**–**d**) OM microstructures of the A356 base material, transition zone, and sticked additive manufacturing layer (SAML) at the consumable rod tip. Reproduced with copyright permission from Springer nature [20].

While friction stir processing (FSP) and the AFS-D are different processes used for various applications, both methods involve similar thermomechanical phenomena. Therefore, one can see many similarities between the two processes concerning microstructure development. The friction-based processes contribute to the increase of the material temperature in the stir deposition region from 0.6 to 0.9 of the melting point of the processed material, which is sufficient for recrystallization via severe plastic deformation through the deposition process [28,34]. To study the A356 consumable rod’s microstructure, it was sectioned vertically, and three points were taken along its length, starting at the first point on the nearest tip to the substrate, going up to the transition zone (heat affected zone), and ending by the base metal, as seen in Figure 9a. The remainder of the consumable rod and the produced AMPs were prepared with the same steps and methods for the microstructure investigation. Figure 9b–d shows the microstructure of the used consumable rod in three different regions. It can be observed that the sticked additive manufacturing layer (SAML) at the consumable rod tip (Figure 9a) shows an equiaxed grain refining microstructure, indicating recrystallization achievement (Figure 9d). This zone is closed to the stir zone in the friction stir welding and processing. The material of the consumable rod recrystallizes before it is transferred to the substrate. Furthermore, this recrystallized material continues to deform during the building of the friction stir deposited layers. Near the consumable rod tip, a thermomechanically affected zone appeared (Figure 9c). In this region, the material suffers from plastic deformation and heat but is insufficient to promote recrystallized grains. Only the heating effect reaches the above region, and it may form a heat-affected zone followed by the base material (Figure 9b).

**Figure 10:**
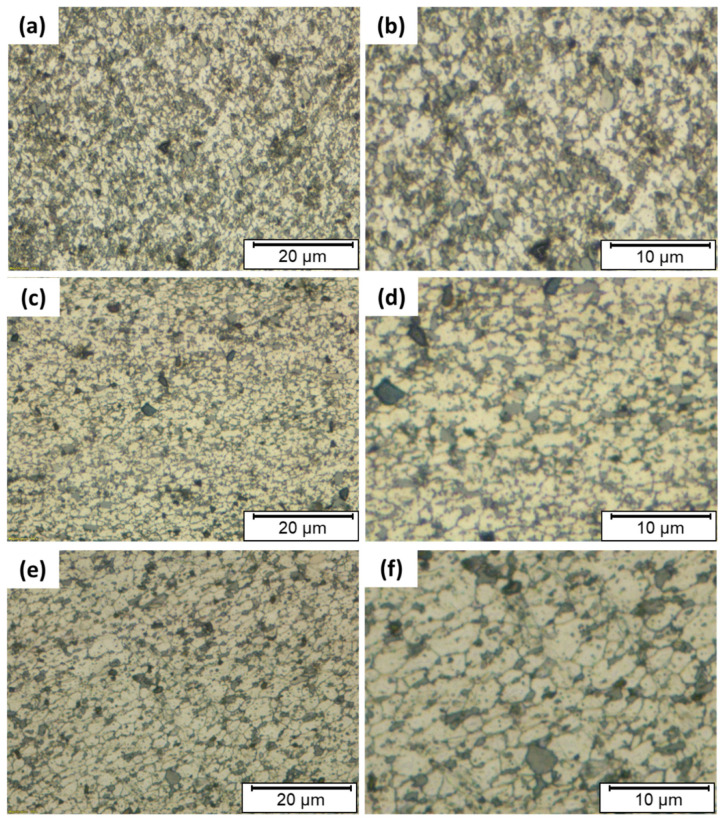
Micrographs of AMPs produced at a constant rotation speed of 1200 rpm and different feeding speeds of (**a**) 3 mm/min, (**c**) 4 mm/min, (**e**) 5 mm/min. Figures (**b**,**d**,**f**) are the higher magnifications of (**a**,**c**,**e**), respectively.

**Figure 11:**
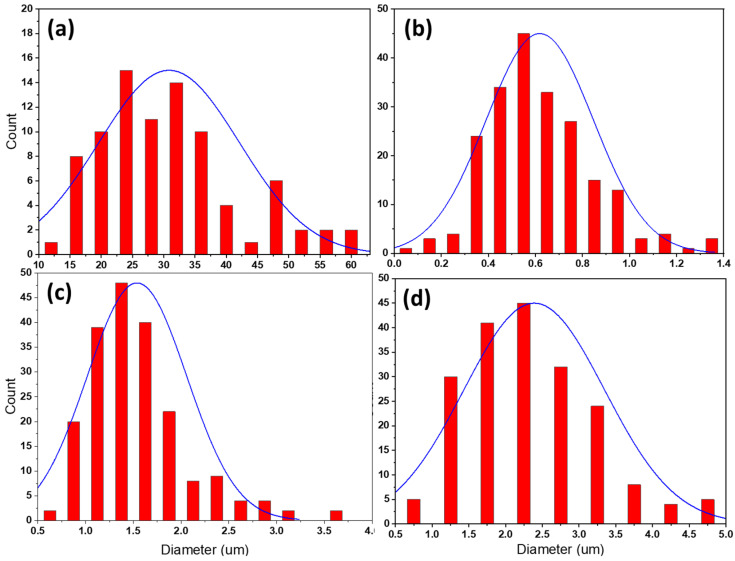
Grain size distribution histograms of (**a**) as-cast A356 alloy and AMPs deposited at a constant rotation speed of 1200 rpm and different feeding speeds of (**b**) 3 mm/min, (**c**) 4 mm/min, and (**d**) 5 mm/min.

Figure 10 shoes optical micrographs of AMPs friction stir deposited at 1200 rpm rotational speed using different feeding speeds of 3 mm/min (Figure 10a,b), 4 mm/min (Figure 10c,d), and 5 mm/min (Figure 10e,f). It is obvious that the dendritic microstructure of the as-cast A356 alloy was totally absent after the FSD. In addition, significantly fine grain structure can be observed. These microstructural features that formed after AFSD can be attributed to the severe plastic deformation at high temperatures experienced during this process. This led to dynamic recrystallization and complex material mixing during the FSD process [1,13,41]. The region of the stir zone during the deposition process experienced a combination of thermal cycle and extensive plastic deformation resulting in an extra-fine microstructure in all the produced AMPs (Figure 10) compared to the dendritic coarse grain of α-Al in the as-cast A356 alloy (Figure 8). This is a characteristic for the friction stir processed materials [42,43]. Previous works [3,4] reported that friction deposited material undergoes continuous dynamic recrystallization and develops very fine equiaxed grains and refined precipitates. Furthermore, the stirring action of the consumable as-cast A356 tool during the AFS-D resulted in the breakup of the coarse primary Si particles, the coarse needle-type eutectic Si, and the intermetallics, to very fine particles. It should also be mentioned that the entire body of the built material was free of porosities, as confirmed in Figure 10. The stirring action of the consumable rod eliminated casting porosities or shrinkage cavities (Figure 9a) by closing them. The AFS-D parameters [20,34,44], had a pronounced effect on the microstructure of the built materials due to the intensified stirring effect during the deposition process. Feeding speed as a processing parameter showed a significant influence on the grain size microstructure of the deposited materials at a constant rotation speed of 1200 rpm, as shown in Figure 10a–f. Figure 11a–d illustrates the grain size distribution histograms of the as-cast A356 and the produced AMPs at different feeding speeds of 3, 4, and 5 mm/min. It can be noted that the mean values of the grain size of the produced AMPs at the feeding speeds of 3, 4, and 5 mm/min were 0.62 ± 0.1, 1.54 ± 0.2, and 2.40 ± 0.15 µm, respectively, compared to the grain size value of 30.85 ± 2 for the as-cast alloy. The AFS-D process affected the range of the grain size for the AMPs compared to the base material. The as-cast A356 grain size ranged from 10 to 60 µm (Figure 11a). After the AFS-D, the minimum grain size range (0.1–1.4 µm) (Figure 11b) was obtained for the AMP deposited at 3 mm/min. The maximum grain size range (0.5–5 µm) was obtained for the AMP produced at 5 mm/min (Figure 11d). This increase in grain size may be ascribed to grain growth as a result of extra heat input with increasing the feed speed.

**Figure 12:**
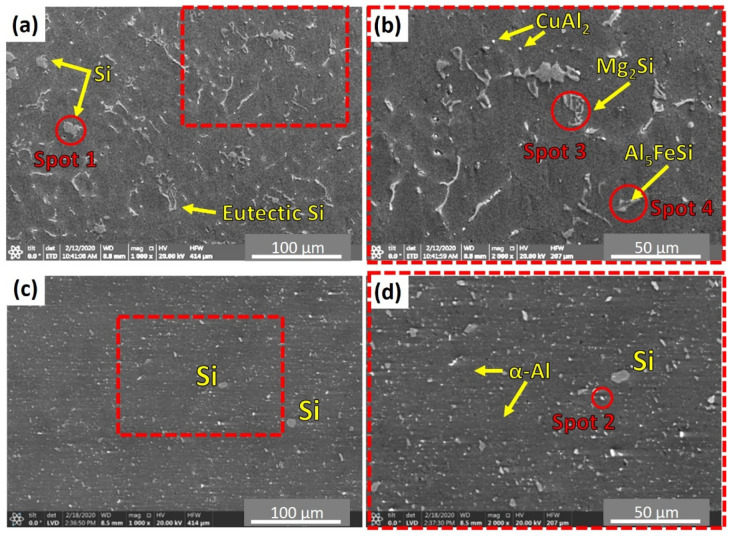
SEM micrographs for the as cast alloy (**a**) low magnification and (**b**) higher magnification, for the AMP deposited at 3 mm/min and 1200 rpm (**c**) low magnification and (**d**) higher mag nification. Note: (**a**,**b**) are SE, while (**c**,**d**) are BSE.

**Figure 13:**
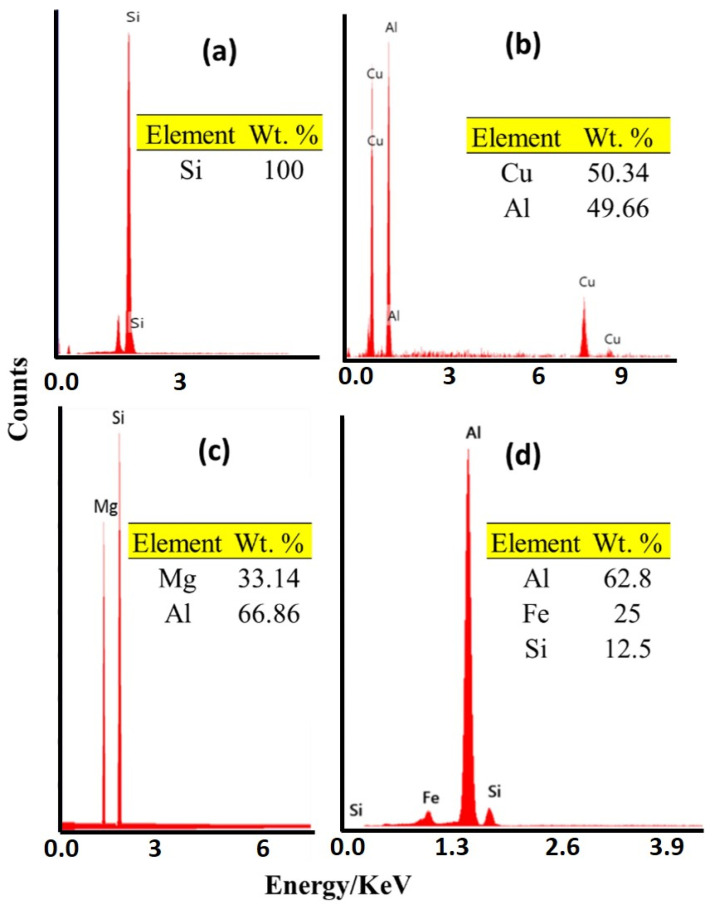
(**a**–**d**) Illustrates the EDS spot analysis for points 1, 2, 3, and 4 indicated on the SEM micrographs in Figure 12, respectively.

To deeply study the effect of feeding speed on the microstructure features of the AFS-D materials, SEM was used in addition to OM. Figure 12 shows SEM micrographs of the as-cast A356 (Figure 12a,b) and the AMP produced after continuous multi-layers of deposited material at 3 mm/min (Figure 12c,d). The SEM micrographs distinctly show the significant favorable influence of AFSD on the morphology and the distribution of eutectic-Si. Figure 13 shows the EDS spot analysis at the positions indicated on Figure 12. The presence of primary Si plates can be seen in Figure 13a, CuAl_2_ presence can be seen in Figure 13b, Mg_2_Si presence can be seen in Figure 13c, and Al_5_FeSi can be seen in the Figure 13d intermetallics. It is essential to say that the size of the α-Al grains was extremely difficult to determine. The grain boundaries of α-Al cannot be easily distinguished after the friction stir deposition process, as seen in Figure 12c,d. These microstructure features confirmed the data obtained by OM. The AFS-D has a thermomechanical zone like the stir zone in FSP [45,46]. SEM investigation by Choi et al. [47] showed that the plate-like Si particles were broken into slightly finer particles by the action of tool stirring during the FSP for A356. Ma et al. [27] studied the influence of friction stir processing (FSP) on the microstructure of as-cast A356. They reported that the FSP refines and homogenizes the as-cast microstructure, eliminates shrinkage porosity, and promotes a microstructure with fine Si particles.

#### 3.2.3. Hardness Results

**Figure 14:**
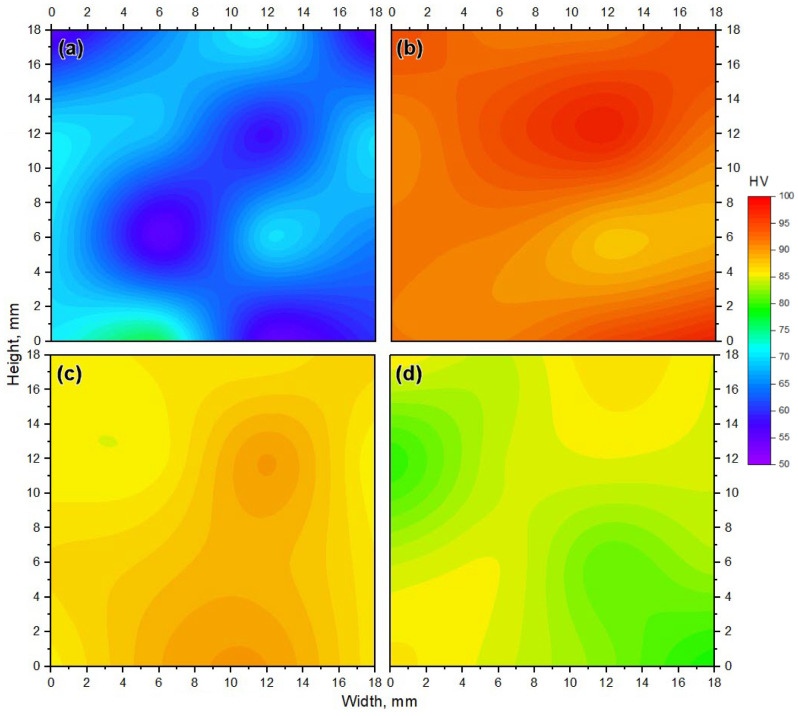
Hardness distribution map for the as cast rod cross section (**a**), and the AMP fabricated parts using 1200 rpm and feeding rates of (**b**) 3 mm/min, (**c**) 4 mm/min and (**d**) 5 mm/min.

**Figure 15:**
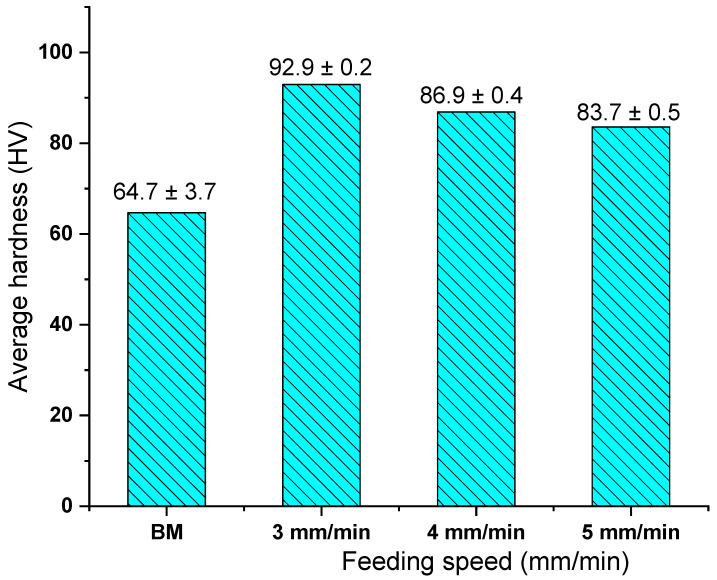
The average hardness values of as-cast A356 and AMPs deposited at a 1200 rpm rotation speed and feeding rates of 3, 4, and 5 mm/min.

Figure 14 shows the hardness map of the as-cast A356 and AMPs deposited at a constant rotation speed of 1200 rpm and various feeding speeds 3, 4, and 5 mm/min. It can be seen from Figure 14a that the as-cast material has a wide range of hardness, from about 50 to 75 HV. The as-cast A356 has a hypoeutectic Al-7.3Si microstructure. The α-Al matrix phase, which is softer than the primary free Si phase and the eutectic Si phase, occupies a large volume fraction. When the indenter of the hardness tester is applied in the α-Al phase, the hardness value is about 50 HV, if it is applied toward the Al-Si eutectic phase or primary Si plates, the hardness value may rise to be around 75 HV. These scattered values in hardness measurements are related to the possibilities of the indenter location in the soft or the hard phase through the surface of the measured as-cast specimen. The same results are reported by [47,48] as they concluded that the cast Al-Si alloy hardness values depended upon the area measured by the hardness indenter. This variation in hardness value confirmed the microstructure features of the as-cast A356, as seen in Figure 8 and Figure 12a,b. However, the hardness maps in Figure 14b–d of the AMPs produced at different feeding speeds of 3, 4, and 5 mm/min, respectively, show more uniform hardness values than those given by the as-cast material, due to the finer and more uniformly dispersed intermetallics, primary Si, and eutectic Si particles, as seen in Figure 10 and Figure 12c,d. The hardness values of the deposited materials were enhanced in all AMPs as compared to the as-cast material, as seen in Figure 15. This may be ascribed to various factors such as the removal of porosity and the enhancement of the microstructures in terms of the reduction of grain size and the fineness and distribution of the eutectic Si, primary Si, and intermetallic particles in the deposited friction stir zone. The maximum hardness value (92.9 ± 0.2) of the AMPs processed at a rotational of 1200 rpm was attained at a 3 mm/min feeding speed, as given in Figure 15. According to the Orowan strengthening mechanism, the reduction of grain size with the presence of particles leads to hardness improvement in the stir zone [49].

## 4. Conclusions

In the current work, friction stir deposition of as-cast hypoeutectic A356 Al alloy was investigated. Solid state additively manufactured parts at the different feeding speeds of 3, 4, and 5 mm/min and a constant rotational speed of 1200 rpm were characterized in terms of macrostructure, microstructure, and hardness distribution. Based on the obtained results, the following conclusions can be drawn:

1. The suggested AFS-D process successfully fabricated sound continuous multilayered A356 AMPs without any physical discontinuities or interfacial defects between the building layers across the vertical direction.
2. The diameter/height ratio of the fabricated AMPs increases with increasing feeding speed at the applied constant rotation rate.
3. The grain refining of the A356 AMPs deposited at feeding rates of 3, 4, and 5 mm/min was 97.9, 95.0, and 92.2, respectively, compared to the grain microstructure of the as-cast material.
4. The enhancement percentage in the hardness of the fabricated A356 AMPs attained 43.6, 34.3 and 29.7% for materials deposited at feeding speeds of 3, 4 and 5 mm/min, respectively, over the as-cast material hardness.

## Figures and Tables

**Table 1:** The nominal chemical composition of as-cast A356 alloy (in wt.%).

Si	Cu	Fe	Mg	Zn	Mn	Ni	Sn	Cr	Ti	Bi	Al
7.3	1.83	1.02	0.35	0.20	0.17	0.10	0.08	0.05	0.04	0.03	Balance

## Data Availability

The data presented in this study are available on request from the corresponding author. The data are not publicly available due to the extremely large size.
